# Aging and “Age-Related” Diseases - What Is the Relation?

**DOI:** 10.14336/AD.2024.0570

**Published:** 2025-06-28

**Authors:** Wolfgang Kopp

**Affiliations:** Retired head of the Diagnostikzentrum Graz, Mariatrosterstrasse 41, 8043 Graz, Austria

**Keywords:** Western diet and smoking, hyperinsulinemia and insulin resistance, subclinical inflammation, sympathetic nervous system, renin-angiotensin-aldosterone system, oxidative stress and mitochondrial dysfunction

## Abstract

The study explores the intricate relationship between aging and the development of noncommunicable diseases [NCDs], focusing on whether these diseases are inevitable consequences of aging or primarily driven by lifestyle factors. By examining epidemiological data, particularly from hunter-gatherer societies, the study highlights that many NCDs prevalent in modern populations are rare in these societies, suggesting a significant influence of lifestyle choices. It delves into the mechanisms through which poor diet, smoking, and other lifestyle factors contribute to systemic physiological imbalances, characterized by oxidative stress, insulin resistance and hyperinsulinemia, and dysregulation of the sympathetic nervous system, the renin-angiotensin-aldosterone system, and the immune system. The interplay between this pattern and individual factors such as genetic susceptibility, biological variability, epigenetic changes and the microbiome is proposed to play a crucial role in the development of a range of age-related NCDs. Modified biomolecules such as oxysterols and advanced glycation end products also contribute to their development. Specific diseases such as benign prostatic hyperplasia, Parkinson’s disease, glaucoma and osteoarthritis are analyzed to illustrate these mechanisms. The study concludes that while aging contributes to the risk of NCDs, lifestyle factors play a crucial role, offering potential avenues for prevention and intervention through healthier living practices. One possible approach could be to try to restore the physiological balance, e.g. through dietary measures [e.g. Mediterranean diet, Okinawan diet or Paleolithic diet] in conjunction with [a combination of] pharmacological interventions and other lifestyle changes.

## 1. Introduction

Aging is a natural, gradual and irreversible decline in physiological homeostasis and organismal fitness as a result of entropy in the cells, tissues, and organs of living organisms. It is associated with a significantly increased risk of numerous chronic and noncommunicable diseases [NCD], leading to reduced quality of life, increased healthcare costs and premature death worldwide [1]. As life expectancy has increased as a result of improved medical care, vaccination and hygiene [2], so has the incidence of chronic “age-related” NCDs. These can be roughly divided into cardiovascular diseases (CVD), neurodegenerative diseases, cancer, disorders of the immune system, eye diseases and musculoskeletal disorders. Among the most common age-related diseases are coronary artery disease, hypertension, heart failure, type II diabetes mellitus (T2DM), cancers, Alzheimer's disease, Parkinson's disease, dementia, chronic obstructive pulmonary disease (COPD), osteoporosis, osteoarthritis (OA), glaucoma and age-related macular degeneration. Each year, an estimated 41 million people die from NCDs, accounting for about 70% of all deaths worldwide. About 17 million of these are younger than 70 years and are classified as premature deaths [3].

Although many of these diseases can occur at a younger age, their incidence increases markedly with age, so a possible mechanistic link between aging and old age is being considered [4]. There are a number of theories to explain the development of these diseases as a result of the aging process [5]. Among the best known are the free radical theory of aging, later renamed the oxidative stress theory of diseases. This theory states that the aging process is due to a progressive accumulation of harmful changes caused primarily by oxidative stress (OxS), which damages macromolecules (lipids, DNA, and proteins) and leads to progressive deterioration of cellular functions and development of degenerative diseases [6]. An extension of the free radical theory of aging, the mitochondrial theory, states that somatic mutations of mitochondrial DNA (mDNA) cause mitochondrial dysfunction, which in turn increases the production of reactive oxygen species (ROS) and OxS in the form of a vicious cycle. Progressive accumulation of dysfunctional mitochondria and oxidative damage with age is thought to play a key role in the aging process [7]. A further development of the above theories, the oxidative-inflammatory theory, states that age-related chronic OxS negatively affect the cells of regulatory systems such as the nervous, endocrine and immune systems in particular. Dysregulation of the immune system causes an inflammatory state which, together with chronic OxS, increases age-related morbidity and mortality [8]. Research into the biology of aging has accelerated considerably in recent decades. A new field of research - geroscience - focuses on understanding the biological mechanisms and identifying common molecular and cellular pathways underlying the aging process and age-related diseases. The aim is to develop therapeutic strategies that alter the course of aging, extend a healthy lifespan and reduce the incidence of age-related chronic diseases by targeting the aging process itself. Key processes and pathways being studied in geroscience include cellular senescence, inflammation, mitochondrial dysfunction and genomic instability [9, 10]. It has been proposed that aging and age-related diseases should be considered as parts of a continuum, since they share, at least in part, some fundamental molecular and cellular changes defined by the hallmarks of aging [1, 11].

However, there are also opposing views on how aging and these chronic diseases are related, and in particular, whether age or the aging process is causally responsible for these diseases [12, 13]. According to Hayflick [13], the aging process does not share common characteristics with any particular disease and is not a disease per se, but increases susceptibility to disease.

To better understand the development of age-related diseases, it is also necessary to identify molecules and families of molecules that may play an important role in various aspects of the aging process and in the development of age-related diseases, such as oxysterols and advanced glycation end products (AGEs). Oxysterols are oxygenated derivatives of cholesterol that occur naturally in the body. In conjunction with inflammation and OxS, excessive amounts of free cholesterol are prone to autoxidation to oxysterols. Some oxysterols such as 7-ketocholesterol (7KC) and 7β-hydroxycholesterol induce inflammation and can significantly stimulate ROS production and apoptosis, and damage organelles such as mitochondria, the endoplasmic reticulum, peroxisomes and lysosomes [14]. 7KC is mainly produced by the autoxidation of cholesterol, but can also be obtained enzymatically. The autoxidation of cholesterol takes place via type I or type II mechanisms. Type I autoxidation is triggered by free radicals; type II autoxidation occurs stoichiometrically by non-radical highly reactive oxygen species such as singlet oxygen, HOCl and ozone [15]. 7KC plays an important role in the pathophysiology of many age-related diseases such as atherosclerosis, Alzheimer’s disease, T2DM, age-related macular degeneration, cataracts, osteoporosis and cancer [14, 16]. Oxysterols, in particular 7KC, 25-hydroxycholesterol and 27-hydroxycholesterol are involved in virtually all stages of the atherogenic process, from endothelial dysfunction and vascular inflammation to the formation of fatty streaks, plaques and plaque rupture [16]. Oxysterols such as 24S-hydroxycholesterol, 27-hydroxycholesterol, 7KC, and 25-hydroxycholesterol play important roles in the pathophysiology of Alzheimer’s disease. They contribute to the disease process through mechanisms involving OxS, inflammation, Aβ production and aggregation, and τ hyperphosphorylation, leading to the formation of neurofibrillary tangles [17]. Diet, heat, cooking methods and the consumption of highly processed foods also have a significant influence on the production of oxysterols in the body [see section 4.1.1.1].

AGEs are a group of molecules that are produced by the non-enzymatic glycation of proteins, lipids and nucleic acids. AGEs are formed endogenously in the human body or come from exogenous sources such as food and cigarette smoke. Exogenous AGEs are present in large quantities in the modern Western diet, primarily due to ultra-processed foods and high-temperature cooking techniques [19] (see also section 4.1.1.1). A persistent hyperglycemic state is a primary source of OxS and is responsible for the increased endogenous production of AGEs in T2DM. This contributes to the higher AGE levels observed in diabetics. Through their major cellular receptor, RAGE, they can induce inflammation, OxS, mitochondrial dysfunction, insulin resistance (IR), disruption of metabolic homeostasis and epigenetic changes, thus playing an important role in the development and progression of T2DM and its complications [18]. AGEs are also associated with other NCDs, e.g. chronic neurodegenerative diseases such as Alzheimer's and Parkinson's, eye diseases and CVDs [20, 21].

## 2. Age-related diseases - an inevitable phenomenon?

From an epidemiological perspective, aging is a significant risk factor for the development of numerous NCDs, such as CVD, hypertension, T2DM, cancer, Alzheimer's disease, Parkinson's disease, COPD, osteoporosis, osteoarthritis, glaucoma and age-related macular degeneration. All of these diseases are chronic in nature, and many are influenced by lifestyle factors such as diet, physical activity, smoking and alcohol consumption. Genetic factors can influence the susceptibility to these diseases [22]. All of these diseases significantly affect the quality of life of those affected and contribute significantly to the burden on the healthcare system. They often occur together, which is known as co-morbidity. Almost 95% of adults aged 60 and over in Western societies have at least one chronic disease, and almost 80% have two or more [23]. Given these statistics, it seems inevitable that chronic diseases will occur as we age. But is aging the primary factor behind the development of these diseases? If, as is generally recognized, aging is a universal process that occurs in most living organisms, from single-celled organisms to complex multicellular organisms such as humans [5, 13, 24], then the development of chronic diseases as part of the aging process should also be a universal phenomenon that affects the entire human population. However, if there are societies whose members remain virtually free of these diseases into old age, this would cast considerable doubt on this assumption.

## 3. Lessons from hunter-gatherers (HG) and other small-scale foraging-based populations

Civilization diseases such as coronary heart disease, obesity, hypertension, T2DM and other NCDs are rare or virtually non-existent in contemporary HG and other foraging-based populations scattered around the world, in the Arctic, savannah, desert and rainforests. Historical and archaeological evidence also suggests that HGs remain largely free of signs and symptoms of chronic NCDs [25-32]. The popular objection that HG generally do not live long enough to develop NCDs is not accurate. The *average* life expectancy in HG societies is lower than in most acculturated societies today due to higher infant and child mortality rates and lack of medical care. However, post-reproductive longevity is a robust feature of HGs. Once these people reach adulthood, their life expectancy is comparable to that of modern populations. A compilation of ethnographic data on HG societies shows that the average modal age of death for adults is 72 decades, with a range of 68-78 years. Causes of death are mainly violence, accidents and infections. NCDs are rare or virtually non-existent [33]. This raises the question of how old the Western population would grow without the blessings of modern medicine. HGs and subsistence farmers are characterized by remarkable cardiovascular health. Studies of HG communities in Central and South America, Africa and Asia, which have been little or not at all influenced by modern civilization, found low blood pressure levels, regardless of gender or age. While 70% of adults aged ≥65 years in the US have hypertension [34], the prevalence of hypertension was between 0 and 10% for most of them [31, 32, 35]. The Tsimane, forager-horticulturalists with a mixed hunter-gatherer and subsistence farming economy living in the Amazon rainforest, are a representative example, as these people have been the subject of intensive research for several decades [31]. Studies have shown that the prevalence of coronary heart disease, hypertension and T2DM among the Tsimane is minimal, and the age-related increase in average blood pressure, which is characteristic of modern populations, is also minimal, if at all [36, 37]. In a study by Kaplan et al [31], 85% [596 of 705 Tsimane] had no coronary atherosclerosis. Among those aged 75 years and older, 65% had a calcium score of 0 and only 8% had calcium scores of 100 or more. CVDs account for a negligible proportion of deaths in these populations, even in adults over 60 years of age [33].

Low serum insulin levels and persistently high insulin sensitivity into old age are also characteristic of HG, as studies have shown, for example, in the Kitava in Papua New Guinea [38], the Aborigines in northern Australia [39], the Hadza, an HG population in northern Tanzania [32] or non-acculturated Indian tribes in Brazil [40]. Tsimani also have normal blood glucose levels, with <1% of adults having elevated morning fasting blood glucose levels [31]. T2DM is rare in these small-scale populations. Prevalence data from 11 populations of HGs, subsistence farmers and pastoralists showed an average prevalence of 1 % [range: 0.0-2.0 %] [29].

However, with the adoption of a Western lifestyle, especially with the switch to a WD, there is inevitably a dramatic increase in IR and hyperinsulinemia, and high blood pressure, arteriosclerosis, T2DM, obesity and other diseases of civilization become commonplace [29, 38, 39, 41-43]. On the other hand, a return to an ancestral diet is associated with significant improvement in IR and fasting insulin levels as well as glucose control and lipid profiles [39, 44-47].

Since the health profiles of these different ethnic groups are so similar, it is obvious that it is the exposome rather than the genes that keep people in these small-scale societies so healthy into old age. The exposome encompasses all environmental influences to which a person is exposed over the course of a lifetime, including diet, physical activity, pollutants and other lifestyle or environmental factors that have a profound effect on health. Significant differences in the exposome between HG societies and people from the modern world may contribute to the development of NCDs. Probably most importantly, there are significant differences between the HG diet and the WD diet. The HG diet consists mainly of game, fish and low-glycemic/low-insulinemic plant foods, such as roots, tubers, wild herbs, berries, nuts, vegetables and fruit, while high-glycemic foodstuff such as refined grains, corn, potatoes, sugars and dairy products are absent [milk and some other dairy products trigger excessive insulin secretion despite a low glycemic index [a kinetic parameter that reflects the ability of a food to raise blood sugar levels] and can cause IR. The proteins in dairy products, especially whey, appear to stimulate this high insulin secretion [48, 49]. The spectrum ranges from a predominantly animal based diet [like the Inuit] to a predominantly plant-based diet (like the !Kung San and the Aka and Efe Pygmies), with many gradations in between.

The WD has spread worldwide and has a significant impact on human health. It is characterized by high levels of high-glycemic/high-insulinogenic carbohydrates such as refined grains, corn, potatoes and sugars, especially glucose, fructose, sucrose and corn-derived fructose syrup, and low levels of fruits, vegetables and fibers. Sugar is consumed in large quantities, especially in soft drinks, but also in the form of sweets and as an additive in bakeries and ready meals. Currently, 85% of the cereals consumed in the US diet are highly processed refined grains [41]. A high glycemic load (GL]) (the multiplication product of carbohydrate amount and glycemic index [50]) is a key feature of WD that plays a crucial role in the development of lifestyle diseases [27, 28, 51, 52]. Indeed, a meta-analysis of 37 prospective cohort studies provides high-level evidence that diets with a high glycemic index, high GL, or both, independently of known confounders, including fiber intake, increases the risk of chronic lifestyle diseases, like T2DM, CVD, breast cancer and gallbladder disease [53]. In addition, the WD contains excessive amounts of omega-6 polyunsaturated fatty acids, primarily through the use of vegetable oil, resulting in an unhealthy omega-6/omega-3 ratio of up to 20:1, whereas the traditional diet has a more balanced ratio. Numerous lines of evidence show that high levels of omega-6 fatty acids promote OxS, oxidised LDL, chronic low-grade inflammation and atherosclerosis [54] and are also associated with the development of a number of other NCDs including T2DM, cancer, obesity, inflammatory bowel disease, Alzheimer's disease and others [54, 55]. In addition, higher levels of physical activity [compared to a more sedentary lifestyle of people in developed countries] are thought to contribute to robust HG health [28, 29]. Numerous studies have shown that physical inactivity is a risk factor for a long list of NCDs, such as CVD, osteoporosis, T2DM, COPD, osteoarthritis, osteoporosis, Parkinson's disease, dementia and others, while regular physical activity can effectively reduce the risk [56, 57]. The lower diversity of the gut microbiota in industrialized countries, which is partly due to a low-fiber diet with a high proportion of processed foods, and the high consumption of antibiotics may also contribute to the development of NCDs [58, 59]. Finally, higher exposure to air pollution, pesticides, industrial chemicals and other environmental toxins is associated with various NCDs, including cancer, respiratory diseases and endocrine disorders [60].

Living and eating habits have changed dramatically worldwide since the agricultural revolution 10,000 years ago and especially since the industrial revolution. In contrast, the human genome has remained largely unchanged [61]. The change in lifestyle has happened too quickly for the human genome to adapt, so humans are still biologically adapted to the environment of their pre-agricultural ancestors [61, 62].

## 4. Lifestyle factors, physiological imbalance and age-related diseases

**Figure 1.**
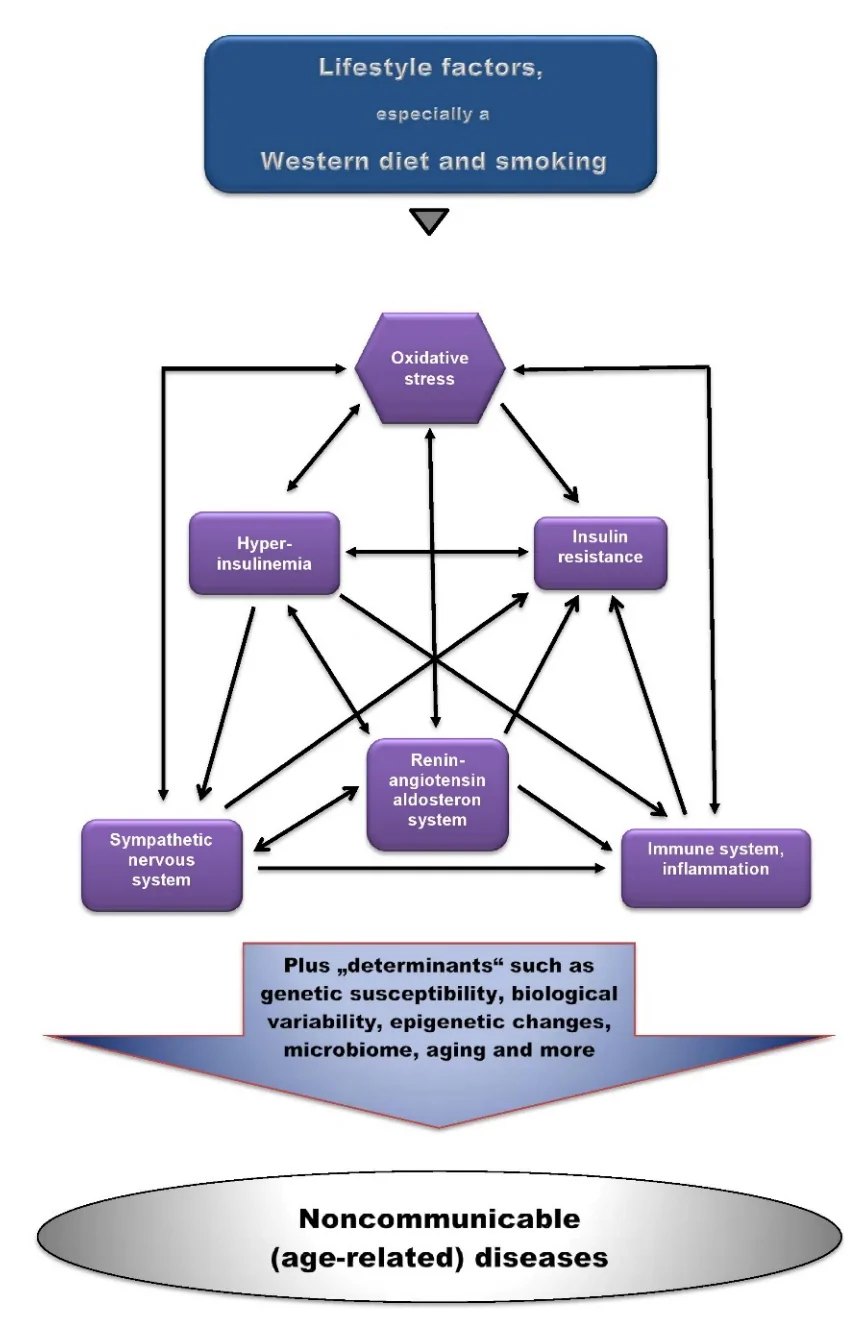
**Pathogenetic model of [age-related] noncommunicable diseases**. Lifestyle factors such as the Western diet and cigarette smoking, can lead to a disturbance of the physiological balance, characterized by OxS, IR and hyperinsulinemia, as well as dysregulation of the SNS, RAAS and immune system. All these factors are closely linked, so that non-physiological changes in one factor can affect the overall balance. It is suggested that the interplay between this pattern and individual factors ["determinants"] such as genetic susceptibility, biological variability, epigenetic changes and the microbiome is primarily responsible for which disease[s] [from a wide range of possibilities] develop in an individual. [Modified from reference 63].

Since the development of NCDs with age is not a global phenomenon and HG only develop NCDs when they adopt a Western lifestyle, it seems unlikely that aging is the cause of these diseases, and it is evident that other mechanisms must be responsible. Recent publications have shown that lifestyle factors such as diet and smoking can cause a profound disruption of physiological balance, which plays a crucial role in the development of a whole range of NCDs such as T2DM, CVDs, cancer, stroke, COPD, arthritis, mental illness and more. A common pathogenetic pattern reflecting this disruption of physiological balance, found in all NCDs examined in these studies, includes dysregulation of glucose and oxidant/antioxidant homeostasis [characterized by OxS, IR and hyperinsulinemia] as well as dysregulation of the sympathetic nervous system (SNS), the renin-angiotensin-aldosterone system (RAAS) and the immune system. All these factors are closely interlinked, so that non-physiological changes in one factor can affect the overall balance (Fig. 1) [63].

The aim of this study is to investigate the relationship between aging and the development of chronic “age-related” NCDs. The main focus is on the relationship between aging and the above-mentioned disruption of physiological balance, and the role of lifestyle factors in the development of NCDs with increasing age.

### 4.1 OxS, mitochondrial dysfunction and aging

ROS and reactive nitrogen species (RNS) play a dual role as constructive and destructive species: on the one hand, ROS and RNS in physiological concentrations are important for the survival of cells, as they regulate biological and physiological processes in the cells as signaling molecules in order to maintain cellular homeostasis. On the other hand, excessive production of reactive species, called OxS, which overrides the intrinsic antioxidant defense system, can disrupt redox signaling and damage macromolecular targets such as lipids, proteins and DNA, including mDNA [64, 65].

Human cells can be exposed to ROS and RNS endogenously and exogenously. Endogenous production of ROS is mainly linked to oxidative phosphorylation (OXPHOS) in mitochondrial complexes I and III of the electron transport chain. Non-mitochondrial sources of ROS include nicotinamide adenine dinucleotide phosphate oxidase (NOX) and NOX homologs, ROS-generating enzymes, and β-oxidation of fatty acids in the peroxisome. Exogenously, ROS and RNS can be produced in response to environmental factors such as tobacco smoking, UV-light, radiation, chemicals, drugs and metals, and industrial pollutants [66, 67].

It is well established that mitochondrial ROS and RNS production increases steadily with age, with mitochondrial dysfunction being one of the most important causes. As we age, mitochondria become highly susceptible to morphological changes that may be associated with reduced function due to oxygen radical damage. The main drivers of mitochondrial defects in aging include chronic production of increased amounts of ROS and RNS, mutations and deletions of mDNA, and impaired clearance of dysfunctional mitochondria through mitophagy [68]. Inflammatory processes also can stimulate the production of ROS as part of the immune response. Conversely, increased levels of ROS can promote inflammation, creating a vicious cycle that contributes to age-related OxS [69]. Cells strive to maintain cellular ROS concentrations in a physiological balance by converting ROS into non-toxic forms through cytosolic antioxidant systems such as catalase, glutathione peroxidase and thioredoxin peroxidase [70]. Mitochondria are protected from OxS by a complex, multilayered network of mitochondrial antioxidant systems, but these protections are not perfect. The mtDNA is particularly vulnerable to oxidative damage because it is located close to the electron transport chain, a major source of ROS, and lacks the protective shields of histones that physically protect nuclear DNA from exogenous OxS [71]. ROS-induced damage to mDNA can lead to impaired function of complex I and/or III, resulting in a vicious cycle with an increase in ROS production and subsequent accumulation of further mtDNA damage [72]. Consistent with this, many studies have shown increased 8-oxo-dG content [one of the most common oxidative lesions] in the mtDNA of aged tissues, as well as increased mitochondrial ROS production in aged cells and tissues [73].

Mitochondria form a dynamic network that undergoes cycles of fission and fusion that regulate the size, shape and distribution of mitochondria in cells. Mitophagy, the selective removal of damaged or dysfunctional mitochondria by autophagic machinery, helps to maintain a healthy pool of functional mitochondria in the cells. Mitochondrial fission and fusion as well as mitophagy play an important role in the regulation of mitochondrial function and quality control. Changes in mitochondrial dynamics and a decrease in mitophagy during aging can lead to an accumulation of damaged or dysfunctional mitochondria. Dysfunctional mitochondria can lead to increased ROS production, which in turn can affect other mitochondria, further exacerbating OxS in a self-reinforcing vicious cycle [74]. In addition to increased reactive species production, aging is associated with a decrease in antioxidant defenses, which contributes significantly to the manifestation of an oxidative stress state [75]. A decline in mitochondrial function and free radical production is associated with the aging process and the development of a number of NCDs.

#### 4.1.1 Influence of lifestyle: poor diet [“Western diet”] and smoking as major drivers of increased ROS production, OxS and mitochondrial dysfunction.

The main task of mitochondria is to regulate cell metabolism and convert nutrients from food into energy. Mitochondria are responsible for the majority of cellular production of adenosine triphosphate (ATP), the universal energy carrier in cells, through OXPHOS. After carbohydrates, fats, and proteins have been broken down into glucose, fatty acids, and amino acids, the tricarboxylic acid cycle produces the reducing agents NADH and FADH2, which donate electrons to the mitochondrial electron transport chain, ultimately leading to the conversion of adenosine diphosphate to ATP. As described above, the WD is characterized by high amounts of high-glycemic/high-insulinogenic carbohydrates and high amounts of fat. Because overeating, frequent snacking, and consumption of sugary soft drinks are now common in westernized societies, a significant portion of the day is spent in the postprandial state which is characterized by a sustained substrate abundance in the circulation [76]. OXPHOS is a demand-driven process in which ATP production is coupled to energy demand. If the supply of electrons exceeds the ATP requirement due to an excess of nutrients, the mitochondrial membrane potential increases, leading to increased production of ROS [77]. An excessive supraphysiologic postprandial rise in blood glucose and blood lipids, termed postprandial dysmetabolism, therefore results in immediate OxS that is directly proportional to the rise in blood glucose and triglycerides after a meal [78]. Artificially induced hyperglycemia peaks by intravenous glucose infusions also significantly increased the formation of free radicals in lean non-diabetics [79]. In addition, nutrient excess can cause mitochondrial dysfunction and increased ROS production by disrupting the mitochondrial life cycle, which is characterized by continuous transitions between elongation (connected state) and fragmentation [disconnected state] [74]. Prolonged nutrient excess disrupts these brief transitions between connected and disconnected mitochondria, leading to fragmentation and eventual mitochondrial damage and an increase in ROS production [80]. High glucose treatment has been reported to cause increased mitochondrial ROS production in both a rat liver cell line and myoblasts, accompanied by dynamic changes in mitochondrial morphology in the form of rapid fragmentation [81]. A high-fat diet rich in saturated fatty acids and enriched with starch, comparable to a WD, also impaired mitochondrial dynamics and function and increased ROS production in an animal study [82].

Furthermore, metabolic inflexibility caused by overeating can lead to chronic OxS. With excessive intake of a high-carbohydrate/high-fat diet, large amounts of substrates [glucose, fat and protein] enter mitochondrial respiration and compete for mitochondrial oxidation, resulting in improper fuel selection and abnormal nutrient partitioning - a phenomenon termed "metabolic inflexibility". In a state of metabolic inflexibility, overfed mitochondria continue to degrade a flood of various incoming carbon substrates, leading to increased production of electron transfer donors (NADH and FADH2) and increased ROS production and OxS [83].

##### 4.1.1.1 Diet

Consistent with the mechanisms described above, animal feeding studies with a WD [84, 85], a high sucrose diet [86] or a high fructose diet [87] resulted in a significant OxS. Fructose, an important component of WD, e.g. in the form of fructose corn syrup or as a component of sucrose, is consumed in enormous quantities, especially as a sweetener in soft drinks and as an additive in ready meals. Cross-sectional clinical studies have shown that diets with a high GL cause significant OxS [88, 89]. Animal feeding studies have demonstrated that high-fat diets [90, 91] and diets high in fat and sugar [92] cause mitochondrial dysfunction.

As mentioned above, 7-ketocholesterol (7KC) is a potent trigger of OxS and organelle dysfunction [mitochondria, peroxisomes, lysosomes, endoplasmic reticulum], inflammation and cell death [14, 15]. Due to the high consumption of animal products, processed foods and whole milk products, the WD is rich in cholesterol, which is susceptible to autoxidation [93]. Oxysterols formed by autoxidation, such as 7KC, are often present in large quantities in industrial foods processed from raw materials with a high cholesterol content [94, 95]. They are also present in significant amounts in foods of animal origin [96] and highly processed industrial foods [97, 95]. Highly processed industrial foods and foods of animal origin often contain increased amounts of oxysterols due to the conditions involved in their preparation, storage, and cooking [98, 99]. Natural cytoprotective molecules include many nutrients found in the Mediterranean diet such as tocopherols, fatty acids and polyphenols. Many Mediterranean oils also reduce the toxicity of this oxysterol [95, 100].

AGEs also play a crucial role in the development of OxS, mitochondrial dysfunction and inflammation and contribute to the development of various chronic diseases. AGEs interact with their specific receptor RAGE. This interaction triggers a cascade of intracellular signaling pathways that promote the production of ROS. The AGE-RAGE interaction also stimulates the release of pro-inflammatory cytokines, which further enhance OxS [101,102]. While hyperglycemia and diabetes are the main endogenous sources of AGEs, the WD contributes significantly to exogenous AGE intake. Ultra-processed foods and some culinary techniques such as frying, grilling, baking or barbecuing are the main sources and drivers of AGEs in the WD [19].

In contrast, the Paleolithic diet [103], the Mediterranean diet [104] and the Okinawa diet [105,106] are associated with low OxS. These diets emphasize natural, unprocessed foods and low-glycemic carbohydrates that are rich in fiber and antioxidants such as phenols. All of these diets are generally associated with a low GL and have anti-inflammatory effects in addition to their antioxidant effects [103-107]. These effects could contribute to the fact that both the Okinawan and Mediterranean diets are associated with longevity and healthy aging as well as a lower risk of chronic diseases [107, 108]. The ketogenic diet, a diet high in fat, moderate in protein and very low in carbohydrates, generally has a very low glycemic load, reduces OxS, improves mitochondrial function and has an anti-inflammatory effect [109].

#### 4.1.2. Smoking

Tobacco smoke contains a complex mixture of toxic chemicals in the particulate and gas phase, e.g. high concentrations of a variety of ROS and RNS such as superoxide, nitric oxide and peroxynitrite. Exposure to cigarette smoke therefore leads to significant OxS [110]. In addition, smoking damages the mitochondrial electron transport chain [111], leading to an imbalance between fusion and fission of mitochondria [112] and mitochondrial dysfunction [111, 113]. In addition, smoking leads to an imbalance in the system of oxidants and antioxidants, resulting in significantly lower levels of antioxidant enzymes [114].

#### 4.1.3. Other sources

Other sources of OxS from lifestyle and the environment include air and water pollution, alcohol, heavy metals or transition metals, industrial solvents and radiation [66, 67] as well as drug abuse, such as cocaine [115] or methamphetamine [116]. Other lifestyle factors such as lack of exercise, poor sleep, chronic stress can indirectly influence mitochondrial function through various pathways, including hormonal and metabolic changes [117, 118].

In summary, mitochondrial dysfunction and OxS are key processes underlying a broad spectrum of chronic diseases. Aging is associated with progressive mitochondrial dysfunction and OxS, where OxS can be both a cause and a consequence of mitochondrial dysfunction. Lifestyle factors may play a crucial role in the development of mitochondrial dysfunction and OxS during aging. In particular, the WD and smoking cause increased ROS production, OxS, and mitochondrial dysfunction. The development of chronic OxS creates a problematic situation as it can lead to IR, hyperinsulinemia and overactivation of the SNS, the RAAS and the immune system [63] (Fig. 1).

### 4.2. IR, hyperinsulinemia and aging

It is well established that glucose tolerance progressively declines with age, explaining the high prevalence of T2DM in the elderly. This decline begins in the third or fourth decade of life and is progressive throughout the entire adult life span. IR and hyperinsulinemia are common in older people, as cohort studies and euglycemic clamp studies have repeatedly shown [119-122]. The mechanism of age-related glucose intolerance is not yet fully understood. A variety of factors are thought to play a role in the development of IR during aging, including mitochondrial dysfunction, increased visceral obesity, decreased physical activity, medication use, intramyocellular lipid accumulation, subclinical inflammation, endoplasmic reticulum stress, decreased autophagy, an overactivated RAAS and genetic factors [123, 124]. Older people are therefore generally more glucose intolerant and insulin resistant, but it remains controversial whether this decline in function is an inevitable consequence of biological aging [125] or the result of other factors such as lifestyle and environment, particularly poor diet and smoking. The most important argument against an inevitable consequence of aging is that the development of IR with age is not a global phenomenon. As mentioned above, low serum insulin levels and persistently excellent insulin sensitivity are characteristic of HG and horticulturalist societies throughout their lives, but only as long as these people maintain their traditional ancestral diet [28, 38, 39, 126, 127]. These people are not genetically immune to the development of IR, because a switch to a WD inevitably leads to a dramatic increase in IR and hyperinsulinemia [29, 128, 129]. On the other hand, a return to a traditional, low-insulinemic diet is associated with a significant improvement in IR and fasting insulin levels [39, 44-47].

#### 4.2.1 Influence of lifestyle - several lines of evidence support a crucial role of lifestyle factors for development of IR.

##### 4.2.1.1 Diet

###### 4.2.1.1.1 Feeding studies

Feeding studies in animals confirm that a high-fat and high-carbohydrate diet, comparable to the WD, plays an important role in the development of IR and glucose intolerance. In an animal study on female Fischer rats, Barnard et al. [130] investigated the effects of a high-fat and high-sugar diet compared to a low-fat and high complex-carbohydrate diet on serum glucose levels and IR. After 24 months, a comparison of the two groups showed that fasting serum insulin was significantly higher and insulin-stimulated glucose transport was significantly lower in the animals reared on a high-fat, high-sugar diet. The authors concluded that diet, not aging per se caused IR. In another animal study, female Fischer rats were fed ad libitum with a high-fat/sucrose diet or a low-fat/complex carbohydrate diet. After 8 weeks, the rats on the high-fat/sucrose diet were insulin resistant and hyperinsulinemic [131]. Similarely, Fischer rats fed ad libitum with a high-fat, refined sugar diet developed hyperinsulinemia and IR before gaining weight. Interestingly, IR developed regardless of whether the fat content of the diet was high [39.5%] or low [9%], suggesting that sucrose is the determining factor [132]. In a long-term feeding study [52 weeks], rats fed an amylopectin-based high-glycemic diet developed IR after 3 months, as measured by intravenous glucose tolerance tests [133]. Feeding studies with a high-fructose diet also caused hyperinsulinemia, IR and OxS [87, 134, 135]. In addition, the WD may contribute to IR through the increased production and intake of AGEs [18].

###### 4.2.1.1.2. ROS and OXS

Increased ROS production and OxS seem to be the link between diet and IR. As described above, nutrient excess and poor diets, like the WD, high-sucrose diets, high-fructose diets and diets with a high GL, produce high levels of ROS and OxS [85-89, 136]. A feeding study with a high-fat diet showed that increased ROS production and OxS preceded the development of IR [137]. Otherwise, induction of IR in cultured cells was blocked when ROS were scavenged [138]. Elevated levels of ROS from various sources, including nutrient excess [139], metabolic inflexibility [83], mitochondrial fission [74], high-fat diets [140], hyperinsulinemia [141], dysregulation of the RAAS [142] and the SNS [143, 144] have been shown to trigger IR. In addition, OxS can trigger endoplasmic reticulum stress which also impairs insulin sensitivity by activating inflammatory pathways [145].

###### 4.2.1.1.3 Hyperinsulinemia

In addition, [diet-induced] hyperinsulinemia can cause IR. For many years, the prevailing view was that visceral obesity was the cause of IR, that IR preceded the development of hyperinsulinemia, and that hyperinsulinemia was merely a compensation for systemic IR. Meanwhile, there is a body of literature that suggests that insulin hypersecretion and hyperinsulinemia precede and cause the development of IR [and obesity] [114, 146-148]. Insulin injections have been used as an experimental model to induce hyperinsulinemia. A study in normal-weight men found that insulin infusion-induced hyperinsulinemia, of the magnitude observed in insulin resistant states such as obesity, produced IR [149]. Another clamp study in healthy individuals also showed that chronic euglycemic hyperinsulinemia over 72 to 96 hours leads to the development of IR and impaired glucose utilization [150]. In an animal model, Cusin et al. [151] demonstrated that short-term hyperinsulinemia [rats were treated with insulin via osmotic minipumps for 3-4 days] led to the development of muscle IR and obesity. Hyperinsulinemia without IR appears to be a relatively common phenomenon that affects a significant proportion of an otherwise healthy population and is a clinically silent harbinger of later development of T2DM over 5-10 years. Between 1970 and 1990, an oral glucose test and simultaneous insulin tests were carried out in a study involving more than 10,000 people. Of the 4185 participants with normal glucose tolerance, just over half (n=2079) had hyperinsulinemia, which was only very slightly associated with obesity [152]. In non-diabetic, normotensive, obese individuals, hyperinsulinemia and insulin hypersecretion was significantly more common than IR [153]. A longitudinal study on insulin dynamics in obese children showed that insulin hypersecretion preceded the development of IR by several years [154]. Also, an abnormal pattern of increased insulin response to normal meals, leading to marked postprandial hyperinsulinemia, was one of the first metabolic changes in a longitudinal study of obese children compared with normal-weight children, followed years later by the development of fasting hyperinsulinemia and IR [155]. In addition, early baseline hyperinsulinemia was the strongest predictor of progression to T2DM in a 24-year longitudinal study [156].

On the one hand, diet-related hyperreactivity of the ß-cells can cause disorders in insulin production. When β-cells are exposed to large amounts of high-glycemic nutrients, a strong postprandial insulin response is triggered [76], which can be further enhanced by the insulinotropic effect of large amounts of saturated fat [157]. The high secretion requirement places a considerable chronic burden on the ß-cells, which can lead to hypertrophy and dysfunction of the ß-cells in the form of hyperreactivity, altered sensitivity to its secretagogue and finally hypersecretion in response to normal meals [158-160]. Indeed, hyperinsulinemia has been shown to be associated with pancreatic islet cell hyperplasia and increased secretory capacity [158, 159, 161]. Female C57BL/6J mice fed a high-fat diet for 12 weeks became obese, hyperinsulinemic, insulin resistant and mildly glucose intolerant. The islet cells of these animals exhibited increased β-cell mass and hypertrophy [162]. Animal studies have shown that pancreatic islet cell hyperplasia precedes and appears to be responsible for the development of insulin insensitivity in the obese mouse, as suppression of hyperplastic islet cells by administration of alloxan or streptozotocin leads to an improvement in insulin sensitivity and hypersecretion *in vivo* [161]. In addition, [diet-related] increased ROS production may also be involved in insulin hypersecretion. As signaling molecules, ROS plays a key role in the regulation and coordination of insulin production. *In vitro* studies performed by Pi et al. [163] showed that ROS [added in the form of hydrogen peroxide or endogenously generated by the addition of diethyl maleate] stimulate insulin secretion in a dose-dependent manner. On the other hand, ROS scavenging prevented both basal and stimulated insulin secretion [163,164]. Recently, Corkey et al. [165] suggested that sustained elevations in both ROS and long-chain acyl-CoA esters lead to basal hypersecretion and basal hyperinsulinemia.

###### 4.2.1.1.4 Inflammation

Although the mechanisms linking inflammation to IR are not yet fully understood, human and animal studies suggest that chronic subclinical inflammation may disrupt normal insulin signaling pathways and promote IR through several mechanisms [166, 167].

##### 4.2.1.2 Cigarette smoking

The negative effects of cigarette smoking on peripheral insulin action are well documented. Numerous studies have shown that cigarette smokers are insulin resistant and hyperinsulinemic compared to non-smokers [168, 169]. A clinical study involving 20 habituell smokers and 20 healthy subjects who were comparable in age, sex and BMI showed that cigarette smoking acutely impairs glucose tolerance in both healthy non-smokers and habitual tobacco smokers, even after the consumption of only three cigarettes [170]. In another study of 7 healthy habitual smokers, Attvall et al. [171] showed that smoking acutely impairs insulin action and leads to IR and hyperinsulinemia. The degree of IR is related to the number of cigarettes/day [172]. Long-term use of nicotine gum is also associated with IR and hyperinsulinemia, as shown in clinical studies by Eliasson et al. [173].

##### 4.2.1.3 Environmental factors

Long time air pollution may contribute to the development of IR [174, 175].

**Figure 2.**
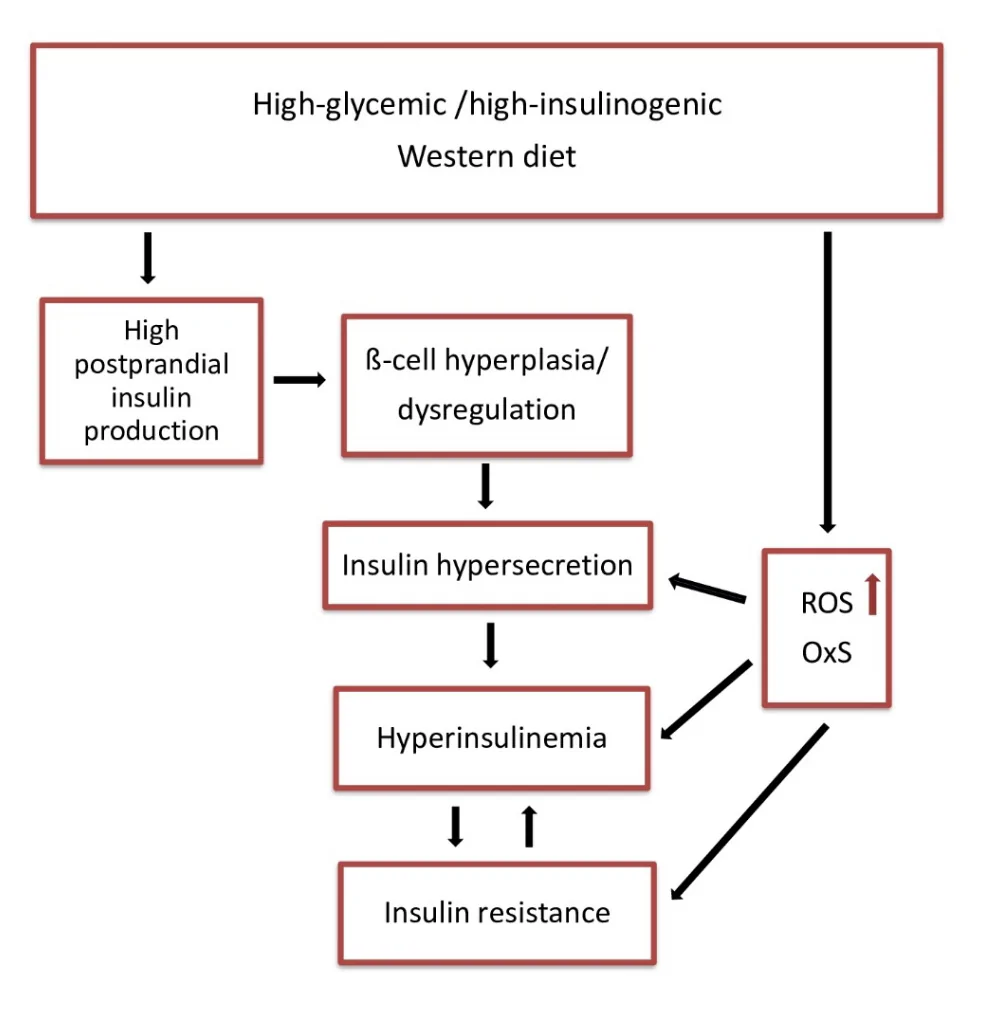
Graphical presentation of the diet-related development of insulin resistance and hyperinsulinemia.

In summary, IR and hyperinsulinemia are common in the elderly and glucose tolerance decreases progressively with age. However, this is not a global phenomenon. The evidence presented shows that a number of lifestyle factors can cause the development of hyperinsulinemia and IR. Unhealthy diets such as the WD and cigarette smoking are widespread in the developed world. Long-term consumption of a high-glycemic/high-insulinogenic WD can cause ß-cell hyperplasia, insulin hypersecretion, hyperinsulinemia, OxS and mitochondrial dysfunction, and eventually the development of IR (Fig. 2).

### 4.3 Aging and dysregulation of the SNS and the RAAS

The SNS and the RAAS are essential physiological systems that play an important role in both normal physiology and pathological conditions. The SNS, one of the two divisions of the autonomic nervous system, is involved in regulating the function and homeostasis of many organ systems in the body via the local release of the catecholamine neurotransmitter norepinephrine (NE) from the sympathetic nerve endings and via the systemic circulation of adrenaline from the adrenal medulla [176].

The RAAS is a hormonal system that plays a crucial role in the regulation of blood pressure, electrolyte and fluid balance. In addition to the classic circulating RAAS with angiotensin II (ANG II) as the main effector, there are also numerous local tissue RAAS that exert autocrine and paracrine effects via the local production of ANG II and can work independently of the circulating RAAS [177, 178], but the circulating RAAS and the paracrine tissue RAAS can also work together in a complementary manner [179].

It is well established that tonic whole-body SNS activity increases with age. Cross-sectional observation studies have shown that plasma NE concentrations increase by 10-15% per decade in adulthood [180, 181], which is primarily due to an increase in NE release at the sympathetic nerve endings and secondarily to a decrease in clearance [182]. The mechanisms underlying the age-related increase in SNS activity are not yet clear, but current evidence points to increased sympathetic drive in the subcortical central nervous system [181, 183]. Two main mechanisms have been proposed to explain the age-related increase in peripheral SNS activity, such as reduced tonic baroreflex inhibition of "normal" central SNS outflow and a primary increase in sympathetic nerve discharge generated by the central nervous system [181].

Numerous *in vivo* and *in vitro* eperiments indicate that aging is associated with increased activity of ANG II, indicating activation of the humoral RAAS. Most studies show an age-related overactivation of the ACE/ANG II/AT1R axis and a reduced antagonistic response mediated by the AT2R as well as the ACE2/ANG [1-7]/MasR axis. Overactivation of the tissue RAAS leads to proliferation, fibrosis, inflammatory response and OxS [178, 184, 185]. Clinical studies have shown that molecules of the ANG II signaling cascade, including ACE, AT1, MCP-1 and MMPs, increase with age in the arterial wall [186]. In addition, long-term ACE inhibition prevented or delayed age-associated vascular structural changes and endothelial dysfunction [187].

#### 4.3.1 Important interrelations

Both the SNS and the RAAS are linked - mainly via positive feedback loops - to other physiological factors (Fig. 1) that play important roles in the development of NCDs [63]. There is a reciprocal feedback loop between the RAAS and the SNS. NE activates ANG II production by stimulating renin secretion in the kidney, while circulating ANG II potentiates NE release from sympathetic nerve endings [188, 189]. There is also a reciprocal relationship between insulin and the SNS: hyperinsulinemia leads to a sustained increase in basal SNS tone via effects on the brain, while chronically elevated SNS tone exacerbates IR and hyperinsulinemia [190, 191]. Insulin activates the SNS in a dose-dependent manner, as evidenced by increased NE plasma levels and micro-neurographic examinations in insulin infusion studies [190]. Studies in obese individuals have shown that increased basal sympathetic nerve activity correlates with the degree of IR, and obesity-induced hyperinsulinemia causes long-term stimulation of the SNS [192]. A positive feedback loop between the activity of the SNS and OxS has been demonstrated [193]. On the one hand, OxS stimulated SNS activity in various experimental models of hypertension [194, 195], while injection of antioxidants led to a decrease in activity [195]. On the other hand, NE increased superoxide production in human peripheral blood mononuclear cells via NOX activation [196]. Finally, overactivation of the RAAS can cause OxS. ANG II is well known to stimulate ROS formation by NADPH oxidases and other prooxidant enzymes and to induce mitochondrial dysfunction, which in turn promotes mitochondrial ROS production [142, 197]. Otherwise, *in vitro* and *in vivo* studies in human aortic smooth muscle cells and in spontaneously hypertensive rats have shown that OxS contributes to the upregulation of vascular AT1R via NFκB activation [198]. Also, OxS was shown to be a direct stimulator of AT1R expression and enhanced ANG II signaling [199].

#### 4.3.2 Influence of lifestyle

##### 4.3.2.1 Diet

Feeding studies in normal humans have shown that high-insulinogenic substrates such as starch and sugar significantly increase SNS activity, which is reflected in a significant increase in plasma NE levels [200], while protein or fat intake has minimal effects on NE levels [201]. In other feeding studies, diets high in carbohydrate and protein were associated with a significant increase in NE levels, while diets high in fat and protein did not alter SNS activity [202, 203].

##### 4.3.2.2 Smoking

Nicotine and particulate matter in tobacco smoke cause a rapid increase in SNS activity, which is reflected in increased plasma catecholamine levels [193, 204]. Cigarette smoke increases ACE protein expression and ANG II levels in tissue, presumably through increased chymase activity, and decreases ACE2 expression in lung tissue [205, 206].

In summary, aging is associated with overactivation of the SNS and the RAAS. Western lifestyle, especially the WD, has a significant impact on SNS activity - and via the above-mentioned feedback loops - on the RAAS, the insulin system and OxS.

### 4.4 Aging and the immune system

Despite the critical need for a lifelong effective and adequate defense against pathogens, the function of the immune system deteriorates with age, impairing both the innate and adaptive immune response. The aging of the immune system is characterized by the paradoxical situation of immunosenescence (insufficiency) and inflammaging (overreaction), which are two sides of the same coin and lead to dysfunction of the immune system. Main features of immunosenescence are a reduced immune response, altered cytokine production, chronic subclinical inflammation, impaired vaccine response, increased risk of autoimmunity, changes in the microbiome and shortened telomeres [207-210]. With increasing age, both the quality and quantity of T and B cell responses decline. The decline in T cell function leads to reduced immune surveillance and a slower response to infections, while changes in B cell function has a negative impact on the production of specific antibodies in response to new pathogens and the diversity of the antibody repertoire. One of the most important causes of age-related changes in the adaptive immune system is the thymic involution, which leads to an almost complete shrinkage of the T cell priming tissue and thus to a reduced production of naive T cells, thereby impairing the immune response [210, 211]. Several factors are thought to contribute to thymus involution, including sex hormones, obesity, autoimmune diseases and infections [212]. A recent study shows that OxS, an important feature of the aging process, can cause thymic involution [213].

Chronic low-grade inflammation, often referred to as “inflammaging”, is a hallmark of immunosenescence. The developmental process involves a dynamic interplay of cytokine-mediated and non-cytokine-mediated processes. Immunosenescence is associated with changes in the production and regulation of various cytokines which play a crucial role in mediating immune responses and maintaining the balance between pro-inflammatory and anti-inflammatory states. This leads to an imbalance between pro-inflammatory and anti-inflammatory cytokines, characterized by an increase in pro-inflammatory cytokines such as interleukin 6 (IL-6), tumor-necrosis factor-α (TNF-α), IL-1, and IL-18, and a relative decrease or insufficient increase in anti-inflammatory cytokines such as IL-10 and transforming growth factor β [207-209]. Other components of the immune system such as neutrophils, macrophages and natural killer cells are also involved and may contribute to inflammation through mechanisms such as the release of ROS and non-cytokine-generating signaling molecules. Additionally, chemokines, although similar to cytokines, play a distinct role by directing immune cell migration to inflammation sites. Lipid-derived mediators such as prostaglandins and leukotrienes also play an important role in inflammation, mediated by enzymes such as cyclooxygenases. The complement system further contributes through a cascade of proteins that enhance the immune response without direct cytokine involvement. Moreover, damage-associated molecular patterns released by damaged cells can initiate immune responses by activating specific receptors [207-209, 214, 215]. Immunosenescence leads to increased susceptibility and severity of infectious diseases and is thought to play an important role in a number of non-communicable age-related diseases such as cancer, CVDs, neurodegenerative diseases such as Alzheimer's and Parkinson's disease, osteoporosis, autoimmunity and others [214-216].

#### 4.4.1 Important interrelations

As outlined in previous sections, aging is associated with OxS, IR and hyperinsulinemia, as well as overactivation of the SNS and the RAAS, all of which can lead to subclinical inflammation [Figure 1], and all of which can occur as a result of poor diet, smoking and other lifestyle and environmental factors. OxS and inflammation are closely linked and interdependent pathophysiological processes. OxS can trigger inflammation by activating signaling pathways that induce the production of pro-inflammatory cytokines and chemokines, while the inflammatory process can trigger OxS [217, 218]. In addition to OxS, hyperinsulinemia [219-221] contributes to the development of chronic subclinical inflammation by impairing the ability of regulatory T cells to suppress inflammatory responses via effects on the AKT/mTOR signaling pathway [222]. Hyperinsulinemic-euglycemic clamp studies in experimental animals have shown that even moderate hyperinsulinemia is sufficient to cause inflammation of adipose tissue [220]. Hyperglycemia and hyperinsulinemia were independently sufficient to trigger an inflammatory response in human chondrocytes by activation of nuclear factor-κB (NF-κB) *in vitro* [221]. Further, dysregulation of the RAAS [223, 224] and the SNS [225, 226] are also causally linked to the development of chronic subclinical inflammation. ANG II acts as a potent proinflammatory mediator by stimulating the AT1R. The inflammatory effects of ANG II are mediated in part by the activation of NF-κB and the production of inflammatory modulators such as TNF-α, IL-1 and IL-6 [227]. For example, ANG II triggers an inflammatory response in human vascular smooth muscle cells by stimulating cytokine production via activation of NF-κB and NOX-mediated ROS production [228]. There is also ample evidence that ANG II has pro-inflammatory effects by increasing the expression of toll-like receptor (TLR) 4 via TLR4-mediated signaling pathways in various organs and cell types such as heart, lung, liver and kidney [229]. Dysregulation of TLRs with NF-κB activation leads to an exaggerated inflammatory response through excessive production of proinflammatory cytokines and chemokines [230, 231]. It is well established that the SNS and its neurotransmitters play a central role in the regulation of inflammation. The immune system is modulated both locally and systemically by the neurotransmitters epinephrine and NE. The chronic activation of the SNS and the release of NE can lead to a dysregulation of the immune system and trigger the production of pro-inflammatory cytokines and increase systemic inflammation [226, 232]. For example, in an animal model of ANG II-mediated hypertension, sympathoexcitation induced NE-mediated T-cell activation and vascular inflammation [233]. Also, a positive correlation was found between sympathetic tone and IL-6 plasma levels [234].

#### 4.4.2. Influence of lifestyle

Lifestyle factors such as the WD and smoking as well as environmental factors may play an important role in the dysregulation of the immune system and the development of subclinical inflammation during aging.

##### 4.4.2.1 Diet

High glucose and insulin levels have been shown to induce excessive OxS and subclinical inflammation in both animal models and humans [235-237]. Accordingly, diets with a high GL and substantial amounts of fat are associated with subclinical inflammation reflected by overproduction of acute-phase proteins such as high-sensitive C-reactive protein and pro-inflammatory cytokines such as IL-6, IL-18 and TNF-α [238, 239]. Although this postprandial inflammatory reaction only lasts a few hours, it occurs several times a day after meals. In an animal study, a WD induced systemic inflammation in Ldlr -/- mice through aberrant activation of the NLRP3 inflammasome, and furthermore reprogrammed immune cells toward inflammatory phenotypes which was no longer detectable in the serum shortly after mice were switched back to a chow diet [240]. In addition to a high GL, excessive amounts of omega-6 polyunsaturated fatty acids and a very high ratio of omega-6 to omega-3, as is typical of today's WD, have a pro-inflammatory effect and promote chronic subclinical inflammation [56]. In addition, increased oxysterol levels can occur in the context of a WD, which in turn activate inflammatory pathways and can contribute to chronic inflammation [14, 94-99]. In contrast, the Paleolithic diet, the ketogenic diet, the Mediterranean diet and the Okinawa diet have an anti-inflammatory effect [103-109]. A low-GL diet also reduced inflammation and tended to increase adiponectin levels, as shown in a randomized crossover study of overweight and obese, but otherwise healthy, adult men and women [241].

##### 4.4.2.2 Smoking

There is ample evidence that cigarette smoking affects both the innate and adaptive immune response [242, 243]. It causes dysregulation of TLRs and NF-κB which disrupts immune homeostasis and leads to an excessive inflammatory response [244, 245]. Cigarette smoking modulates and promotes chronic inflammation by increasing the production of pro-inflammatory cytokines such as TNF-α, IL-1, IL-6 and IL-8 and decreasing the levels of anti-inflammatory cytokines such as IL-10 [246]. In summary, aging is associated with immunosenescence and is accompanied by a chronic inflammatory state which underlies many age-related NCDs. Lifestyle may play an important role in the dysregulation of the immune system and the development of subclinical inflammation during aging. Lifestyle factors, especially WD and cigarette smoking, cause a disruption of physiologic balance leading to immune dysregulation and chronic subclinical inflammation through the production of OxS and hyperinsulinemia, as well as dysregulation of the RAAS and the SNS.

## 5. The most common age-related diseases: do they follow the pattern described above?

As described above, lifestyle factors can disrupt physiological balance over the course of a lifetime, characterized by OxS, IR and hyperinsulinemia as well as dysregulation of the SNS, RAAS and immune system. A previous publication has already shown that this pattern plays an important role in a number of NCDs and suggested that it represents a kind of pathogenetic "toolkit" for the possible development of many [most?] NCDs [63]. To further substantiate this theory, we will investigate whether this also applies to so-called age-related diseases. For this purpose, the most common age-related diseases are to be examined. These include CVDs, cancer, osteoarthritis, COPD, osteoporosis, T2DM, hypertension, Alzheimer's and Parkinson's disease, benign prostatic hyperplasia (BPH) and glaucoma. As the pattern described above has already been demonstrated in a previous publication in CVDs, hypertension. cancer, COPD, osteoporosis, T2DM, hypertension and Alzheimer's disease [63], BPH, osteoarthritis, Parkinson's disease and glaucoma remain to be investigated here.

### 5.1. Benign prostatic hyperplasia [BPH]

BPH, a common condition in older men, is characterized by non-cancerous enlargement of the prostate and is the most important factor in the development of lower urinary tract symptoms. The incidence increases with age, with approximately 50% of men aged 51-60 and up to 90% of men aged 80 and older experiencing symptoms of BPH [247]. BPH is characterized by the appearance of hyperplastic nodules due to increased proliferation of epithelial and stromal cells, especially in the periurethral region and the transition zone [248], as well as increased sympathetic smooth muscle tone [249, 250].

The exact cause of BPH is not fully understood, but it is believed to involve both hormonal and non-hormonal factors. Most interest has been focused on hormones, especially dihydrotestosterone. Age-related hormonal changes, particularly changes in dihydrotestosterone levels and androgen receptor expression in the prostate, as well as chronic inflammation, are thought to play a role in the occurrence and progression of BPH and lower urinary tract symptoms [251]. However, a solid pathogenetic model should provide an explanation for the fact that BPH develops [almost] exclusively in the periurethral region and the transition zone. Disruption of physiology, including OxS, dysregulation of insulin metabolism, SNS and especially of the RAAS, could play a key role in the developmental process and better explain this peculiarity of BPH [252].

#### 5.1.1 OxS

In *vitro* and in *vivo* studies have identified OxS as an important signaling pathway involved in the development of benign prostatic hyperplasia. Patients with BPH have higher urine markers of OxS than age-matched healthy individuals [253]. Human BPH tissue from the transition zone contained significantly higher concentrations of 8-OH-deoxyguanosine, a marker for OxS, than control tissue, and its concentration correlated with prostate weight [254]. A causative role of OxS in BPH was confirmed by a transgenic mouse model with overexpression of NOX4 under the control of the prostate-specific promoter ARR2PB. The transgenic mice exhibited increased oxidative DNA damage in the prostate, increased prostate weight, increased epithelial proliferation, stromal thickening and fibrosis compared to wild-type controls [254].

#### 5.1.2 Hyperinsulinemia and IR

Hyperinsulinemia and IR were found to be independent risk factors for the development of BPH [255, 256]. Several studies have shown that fasting serum insulin levels were significantly higher in men with BPH than in control subjects without BPH [256, 257]. Cross-sectional studies have found a direct correlation between insulin levels and the annual growth rate of BPH [257]. Hyperinsulinemia can directly affect prostate growth through insulin receptor-mediated growth-promoting effects [258] and, due to its structural similarity to insulin-like growth factors (IGF) [259], by enhancing IGF-1 receptor signaling [260]. In *vitro* studies have highlighted the importance of the IGF axis for cell growth in the human prostate [261]. The role of the IGF axis in BPH is also supported by studies showing that the expression of IGF1 receptors is much higher in the periurethral region than in the intermediate and subcapsular regions of BPH tissue. In addition, dihydrotestosterone levels were highest in the periurethral region, suggesting that BPH is promoted by IGF in a hormone-dependent process [262]. In addition, hyperinsulinemia may lead to increased IGF-1 bioavailability due to insulin-mediated changes in IGF binding protein [260].

#### 5.1.3 SNS and RAAS

Smooth muscle cells make up a large part of the prostate. The tone of the smooth muscles in the gland is regulated by the adrenergic nervous system. The prostate is innervated by sympathetic nerves, which release NE when stimulated and cause smooth muscle contractions mediated by α-1-adrenergic receptors [263]. Increased local sympathetic activity and increased smooth muscle tone are characteristic features of BPH and are associated with the pathogenesis of BPH by stimulating the proliferation of non-epithelial prostate cells and thereby influencing the growth of the prostate [249, 250]. Of particular importance is that overactivation of the SNS leads to activation of the RAAS [188, 189]. There is growing evidence that the local RAAS plays an important role in the development of BPH [264]. Expression of ANG II and angiotensin-converting enzyme, a key component of the RAAS, is abnormally elevated in BPH [264, 265]. ANG II is known to stimulate cell proliferation and growth of vascular smooth muscle cells and to increase vascular smooth muscle tone [266]. Similarly, a mitogenic and proliferative effect of ANG II on the stromal compartment of human prostate tissue mediated by AT1 receptors has been demonstrated [267]. The importance of SNS and RAAS overactivity is also illustrated by the fact that AT1Rs are highly concentrated in the periurethral region and in the transition zone, which are most affected by BPH [265, 268].

#### 5.1.4 Immune system/inflammation

BPH may be an immune-inflammatory disease, and inflammation may play an important role in the development and progression of BPH, as clinical studies have shown. Inflammation can influence the development of BPH independently in various ways and can also interact with androgens [269]. A study of 282 patients who underwent surgery for complicated and/or symptomatic BPH found that inflammatory cells infiltrated the BPH tissue in the majority of patients. The international prostate symptom score and prostate volume were significantly higher in patients with high-grade prostatitis [270]. There is increasing evidence that a dysregulated immune system with autoimmune reactions could play a decisive role in BPH [271, 272]. Approximately 90% of prostate immune cells are T lymphocytes. The T-cell activity and associated autoimmune reaction seem to induce epithelial and stromal cell proliferation [269, 273]. However, the contribution of the immune system and inflammation to the development of BPH is not yet fully understood and more research is needed on this aspect.

### 5.2 Osteoarthritis (OA)

OA is a chronic, multifactorial degenerative and mildly inflammatory disease of the synovial joint that affects the entire joint including bone, cartilage, ligaments, fat and synovial tissue and can severely impair the patient's quality of life. It is characterized by progressive articular cartilage degradation, osteophyte formation, synovitis, and subchondral bone sclerosis. OA is the second leading cause of severe chronic disability after CVD, the most common form of arthritis in the U.S., and its incidence and prevalence are increasing [274]. According to estimates, 250 million people worldwide suffer from knee osteoarthritis [275]. OA is often accompanied by numerous comorbidities [276].

The pathogenesis of OA is based on a complex interplay of biomechanical, biochemical and genetic factors. Originally, OA was considered an “almost inevitable consequence of aging” caused by biomechanical factors. Today, OA is no longer considered to be a purely non-inflammatory biomechanical process, but rather an interplay between mechanical damage and chronic subclinical inflammation [277, 278]. While chondrocytes resist proliferation and terminal differentiation in healthy articular cartilage, chondrocytes in diseased cartilage progressively proliferate and develop hypertrophy. Proliferation and hypertrophic differentiation of chondrocytes, the remodelling and mineralization of the extracellular matrix, the penetration of blood vessels and the apoptotic death of chondrocytes play an important role in OA [279]. There is growing evidence that the epigenome also plays an important role and that a combination of epigenetic and genetic modulation may contribute to the development and progression of OA [280].

#### 5.2.1 OxS

OxS and inflammation in chondrocytes and other joint tissues play a critical role in the development and progression of OA. Many studies have shown that the ROS concentration in human OA cartilage and chondrocytes is greatly increased [281, 282]. OxS induces inflammation in joint tissue, which contributes to the breakdown of cartilage and other joint structures. Inflammatory mediators such as IL-1β, TNF-α and IL-6, which are highly expressed in OA joints and actively produced by chondrocytes, synoviocytes, macrophages and osteoblasts, play a crucial role in the degradation of the cartilage matrix. Thus, chondrocytes are both the source as well as the target of proinflammatory cytokines in OA [281]. In addition, OxS significantly accelerates telomere shortening and chondrocyte aging, which impairs redox regulation of mitochondria in cartilage and stimulates ROS production, further exacerbating the disease [283].

#### 5.2.2 IR and hyperinsulinemia

Epidemiological studies have shown that T2DM and obesity are independent risk factors for the development of OA, suggesting a possible involvement of IR in the pathogenesis of OA. The association between OA and T2DM is well established as more than 90% of patients diagnosed with T2DM 2 are obese [284]. IR has been reported to be directly involved in the pathogenesis of OA by increasing the expression level of TNF-α in synovial cells. Synovial fibroblasts respond to TNF-α with increased production of cytokines, growth factor BMP2, and proteinases involved in the development of osteophytes that form near the inflamed synovium [285]. In *vitro* studies using human chondrocytes revealed that hyperinsulinemia and hyperglycemia independently trigger inflammatory responses in human chondrocytes by activating the transcription factor NF-κB, which plays a critical role in OA by inducing the expression of pro-inflammatory and catabolic genes [221].

#### 5.2.3 SNS

Bone and joint tissues are densely innervated by sympathetic nerve fibers [286]. As an important regulatory mediator between the brain and the immune system, the SNS influences inflammatory processes throughout the body through the release of neurotransmitters such as NE. Increased activity of the SNS causes bone resorption in subchondral bone through activation of osteoclasts via RANK-L [287]. Dysregulation of sympathetic signaling may disrupt joint homeostasis, leading to cartilage degradation and OA progression. Neurogenic inflammation mediated by sympathetic nerve fibers has been observed in synovium and periarticular tissues. Aberrant sympathetic signaling may exacerbate pain perception, contributing to the chronic pain experienced by patients [288, 289]. An in *vitro* study using stem cells from synovial adipose tissue suggests that NE suppresses [stem cell-dependent] articular cartilage chondrogenesis and regeneration and thereby contributes to the development of OA [290]. Clinical studies, examining joint destruction and pain intensity in OA patients, have clearly demonstrated that the β2-adrenergic receptor plays a critical role in the progression of OA [291, 292].

#### 5.2.4 RAAS

In recent years, studies revealed that overactivation of the RAAS is involved in the pathogenesis of OA. A local RAAS has been shown to regulate cartilage development and homeostasis. Expression of AT1R and AT2R mRNA was detected in human chondrocytes in an in *vitro* study that included patients with osteoarthritis, rheumatoid arthritis and traumatic fractures [293]. RAAS-related components such as renin, ACE, ANG II, AT1R and AT2R are involved in inflammation and hypertrophic differentiation of chondrocytes [294], key features of OA. ANG II promoted hypertrophic differentiation of chondrocytes and decreased apoptosis of hypertrophic chondrocytes through AT1R activation and increased the expression of genes related to hypertrophic differentiation and cartilage matrix regeneration in a mouse model, while olmesartan, an AT1R blocker, attenuated hypertrophic differentiation [295]. Key components of the tissue-based RAAS, including ACE/Ang II/AT-1R, are also synthesized and active in bone cells, osteoblasts and osteoclasts [296] and human and animal synovial tissue, and their expression levels correlate with the degree of synovial inflammation and the severity of arthritis [294, 297].

#### 5.2.5 Immune system and inflammation

Activation of the innate immune system is crucial for the initiation and maintenance of low-grade inflammation. Once activated, the inflammatory response leads to upregulation of catabolic factors such as proinflammatory cytokines, proteolytic enzymes and chemokines and downregulation of anabolic factors such as anti-inflammatory cytokines and growth factors, contributing to pain and cartilage damage [298, 299]. Immune cells, including macrophages and T lymphocytes, can invade the synovial membrane and cartilage and release pro-inflammatory factors that perpetuate the cycle of inflammation and tissue damage in the joint [299, 300]. Dysregulation of TLRs, particularly TLR2 and TLR4, is thought to contribute to the chronic inflammation and tissue destruction that characterize the disease [301, 302].

### 5.3 Parkinson’s disease (PD)

PD is the second most common neurodegenerative disease after Alzheimer's. Key pathological features include neuroinflammation, degeneration of dopaminergic neurons in the pars compacta of the substantia nigra and striatum, and abnormal deposition of α-synuclein in intracytoplasmic and intraneuritic inclusions known as Lewy bodies and Lewy neurites. A progressive loss of dopaminergic neurons in the substantia nigra leads to the development of motor symptoms such as bradykinesia, tremor and rigidity. Most cases of Parkinson's disease are sporadic, about 10% of cases are inherited, and several genetic mutations have been identified that increase the risk of developing the disease [303]. While the exact cause of Parkinson's disease is not fully understood, it is believed to involve a combination of genetic susceptibility, cellular mechanisms and environmental and lifestyle factors. Clinical and postmortem studies as well as animal models suggest that OxS, mitochondrial dysfunction, a disrupted proteasomal system, impaired autophagy/mitophagy, excitotoxicity and neuroinflammation are key factors in the pathogenesis and progression of this neurodegenerative disorder [304, 305].

#### 5.3.1 OxS and mitochondrial dysfunction

Mitochondrial dysfunction plays an important role in the pathogenesis of Parkinson's disease. Mitochondrial dysfunction in dopaminergic neurons, which are particularly susceptible to energy deficits due to their high metabolic demand, impairs ATP production and calcium buffering, which are crucial for the function of these neurons, and exacerbates OxS [306, 307]. Mitophagy, the selective degradation of mitochondria, is an essential component of mitochondrial homeostasis and critical for neuronal health. Impaired mitophagy and OxS are key pathological features in Parkinson's disease and are associated with accelerated neurodegeneration [307].

#### 5.3.2 IR and hyperinsulinemia

Although there are relatively few studies on the role of IR in the development of Parkinson's disease and a lack of biological evidence in human studies, there is nevertheless some evidence that IR may play an important role. A number of studies suggest that T2DM increases the risk or accelerates the progression of Parkinson's disease and that T2DM and Parkinson's have similar dysregulated signaling pathways, suggesting common underlying pathological mechanisms [308-310]. In a sample of 154 non-diabetic Parkinson's patients, a high prevalence of IR was found, with 58% being insulin resistant, as indicated by abnormal HOMA-IR values [311]. In a recent study, Hong et al. [310] investigated the effects of IR in *vitro*, ex *vivo* and in an in *vivo* MitoPark mouse model of Parkinson's disease. They found that IR promotes the development and progression of PD through mitochondrial dysfunction and increased ROS production and reported abnormal α-synuclein expression and increased degeneration of dopaminergic neurons. IR induced by a high-fat diet in an animal study resulted in impaired nigrostriatal dopamine function, characterized by decreased dopamine release and dopamine clearance, and increased iron deposition in the substantia nigra - hallmarks of Parkinson's neuropathology [312].

#### 5.3.3 RAAS

Regulatory interactions between the dopaminergic system and the RAAS have been identified in the substantia nigra and striatum. The RAAS is involved in the progression of Parkinson's disease by promoting OxS and neuroinflammation [313]. A recent study shows that the dopaminergic neurons that are most susceptible to degeneration in Parkinson's disease are characterized by high expression of the AT1R [314]. Overactivation of the local RAAS exacerbates OxS, inflammatory microglial responses and dopaminergic degeneration, all of which are inhibited by angiotensin receptor blockers and angiotensin-converting enzyme inhibitors [313]. In animal models, dopamine deficiency leads to compensatory overactivation of the local RAAS, which promotes microglial responses and neuronal vulnerability through activation of NADPH oxidase [315].

#### 5.3.4 Immune system/inflammation

There is increasing evidence that inflammation in the brain, which is triggered by dysregulation of the immune system, is associated with the progression of Parkinson's disease. Microglia, the immune cells of the brain, can become overactivated, transforming neuroprotective astrocytes into neurotoxic astrocytes and thus contributing to neuroinflammation and the death of neurons. Neuroinflammation can exacerbate neuronal damage and promote the accumulation of misfolded proteins, further exacerbating neurodegeneration. There is also evidence of peripheral adaptive immune system involvement in PD, suggesting a systemic inflammatory component to the disease [316, 317].

### 5.4 Glaucoma

Glaucoma is a group of neurodegenerative diseases characterized by progressive structural damage, apoptotic death and loss of function of retinal ganglion cells, characteristic optic atrophy and visual field loss. It is one of the leading causes of irreversible blindness in the world. Glaucoma can be broadly divided into three categories: open angle glaucoma, angle closure glaucoma and normal pressure glaucoma, depending on whether the anterior chamber angle is open or not and whether the intraocular pressure is elevated [318]. Increased intraocular pressure is a hallmark characteristic of most forms of glaucoma. It can lead to ischemia, impairment of axonal transport, OxS and inflammation of the optic nerve [319]. Several factors are thought to contribute to the development of glaucoma, including OxS, mitochondrial dysfunction, protein misfolding, excitotoxicity, hypoxic and ischemic phenomena, and alterations in neurotrophin signaling [320, 321].

#### 5.4.1 Mitochondrial dysfunction and OxS

Mitochondrial dysfunction and OxS may contribute to the pathogenesis of glaucoma through multiple interrelated mechanisms, ultimately leading to retinal ganglion cell degeneration and optic nerve damage, the hallmarks of glaucoma. The retina is a part of the central nervous system with a high metabolic rate and a high energy requirement due to its constant activity in detecting and processing light signals. Therefore, retinal cells, especially photoreceptor cells, have a relatively high density of mitochondria compared to many other cell types [322]. This high metabolic activity makes them more susceptible to mitochondrial dysfunction and OxS, which can damage various cellular components such as proteins, lipids and DNA, eventually leading to degeneration of retinal ganglion cells and the optic nerve. Furthermore, mitochondrial dysfunction can lead to insufficient energy production, resulting in cell damage and ultimately death of the retinal ganglion cells. In addition, dysfunctional mitochondria can release pro-apoptotic factors into the cytoplasm, triggering the apoptotic cascade and leading to retinal ganglion cell death [323, 324].

#### 5.4.2 RAAS

Local RAAS have been identified in various tissues, including the retina, which are important for the maintenance of local homeostasis [325]. While the pathologic processes at the molecular level are not yet well understood, the local ocular RAAS appears to play a role in ocular pathology. Dysfunction of the RAAS can lead to neuroinflammation and activation of glial cells, such as astrocytes and microglia, which play important roles in the pathogenesis of glaucoma [326]. ANG II/AT1R signaling can increase OxS and inflammatory responses through the activation of NAD(P)H oxidases [327]. There is some evidence that the ocular RAAS may be involved in aqueous humor formation, outflow and regulation of intraocular pressure, as ANG II has been reported to be able to induce cell proliferation in bovine trabecular meshwork cells and increase collagen synthesis in *vitro* [328]. There is also growing evidence that RAAS inhibition can prevent the progression of various eye diseases, including glaucoma [329].

#### 5.4.3 IR and hyperinsulinemia

Insulin and IGF-1 receptors are abundant in the brain and there is growing evidence that they are involved in various neurodegenerative diseases [330], including glaucoma [331, 332]. Brain IR can lead to impaired neuronal signaling and neuroinflammation, vascular dysfunction with reduced blood flow to the optic nerve and retina, and dysregulation of neurotrophic factors that impair retinal ganglion cell survival and function [333, 334] and is thought to contribute to the development of glaucoma [335]. IR has been shown to correlate positively with increasing intraocular pressure [336]. In an animal study, brain IR induced by a biosynthetic insulin receptor antagonist led to a neurodegenerative phenotype characterized by inflammation, glial activation, apoptosis in the retina and optic nerve, dysfunction of the trabecular meshwork and ciliary body, and increased intraocular pressure [337]. In addition, hyperinsulinemia was found in patients with glaucoma or central retinal vein occlusions compared to the control group [338].

#### 5.4.4 Immune system and inflammation

Increasing evidence suggests that dysregulation of the immune system and neuroinflammation are part of the sequence of pathological events that lead to optic neuropathy. Chronic low-grade inflammation, characterized by the presence of inflammatory mediators and activated immune cells, may contribute to the neurodegenerative process in glaucoma. Inflammatory cytokines, such as TNF-α, IL-6, and IL-1 beta, were elevated in the aqueous humor and tissues of glaucoma patients [339]. OxS is crucial for the triggering and dysregulation of immune activity during glaucomatous degeneration. Inflammation associated with the activation and proliferation of resident glial cells, like astrocytes, Müller cells and microglia, have been described as common features of clinical and experimental glaucoma [321]. Autoantibodies against retinal antigens have been detected in the serum and aqueous humor of glaucoma patients, supporting the notion of autoimmune involvement [340].

## 6. Discussion

The population in developed and developing countries suffers from a large number of chronic NCDs, many of which occur more frequently with increasing senescence and are therefore referred to as “age-related” diseases. As shown above, the development of these diseases is not a universal phenomenon. Numerous studies show that today's HG and other small-scale foraging-based populations around the world remain virtually free of NCDs throughout their lives and into old age, but only as long as these people maintain their way of life, especially their ancestral diet. As outlined above, there are significant differences in the exposome between HG and people from the developed part of the world that may be responsible for this phenomenon. As studies on HGs, as well as numerous studies on humans and animals have shown, the difference between the WD and the ancestral “Paleolithic” diet of HG societies most likely plays a key role in this phenomenon, especially with regard to its effects on metabolism and physiological balance. While the low-glycemic/low-insulinogenic ancestral diet is associated with low OxS, high insulin sensitivity, no significant subclinical inflammation and impact on SNS activity [38, 47, 103, 104, 201], the high-glycemic/high-insulinogenic WD has a strong tendency to disrupt physiological balance and cause OxS, IR, hyperinsulinemia and dysregulation of the SNS, RAAS and immune system. Cigarette smoking causes the same physiological imbalance [63], and drug abuse, air and water pollution, etc. can have a negative impact on physiological balance through the development of OxS (Fig. 1]. It has been suggested that a pattern of OxS, IR, hyperinsulinemia and dysregulation of the SNS, RAAS and immune system provides a pathogenetic basis [“toolkit”] for the development of many [most?] NCDs [63]. However, if the common pathogenetic pattern described above enables the development of numerous NCDs, why do affected individuals develop only one or a few of the many diseases? In this regard, it has been suggested that the decision as to whether and which disease(s) develops in a person is determined by other, individual factors ("determinants") [63]. Individual factors such as genetic susceptibility, biological variability, epigenetic changes and the microbiome must be taken into account. These factors that vary from person to person can be considered as potential vulnerabilities of an organism that may favor the development of certain diseases in the context of a physiological imbalance [341]. Genetic variations such as single nucleotide polymorphisms, copy number variations, insertions and deletions affect a wide range of biological processes, including lipid metabolism, glucose metabolism, bone health, immune response and neuronal function. They can affect how people react to lifestyle and environmental influences and make them susceptible to developing certain diseases [22, 342, 343]. Biological variability refers to the natural differences observed in biological systems among individuals. This includes genetic, molecular, cellular, and physiological variations and can influence susceptibility to disease and its progression [344, 345]. Epigenetic changes such as DNA methylation and histone modification can alter gene expression without altering the underlying DNA sequence and vary from person to person. These changes may be influenced by environmental factors such as WD, cigarette smoking, air pollution, polycyclic aromatic hydrocarbons, pesticides, dioxins and plasticizers [240, 346-348] and may influence disease risk. Finally, the exposome significantly influences the composition and function of the microbiome. The WD, pollutants like air pollution and pesticides, medication, lifestyle factors like smoking and alcohol, chronic stress and aging all can significantly alter the microbiome. The changes disrupt the balance of the intestinal microbiome and promote the growth of pathogenic bacteria, can lead to increased intestinal permeability ("leaky gut") and cause systemic inflammation. These changes in the microbiome can contribute to the development and progression of age-related diseases through mechanisms involving inflammation, immune response, metabolic health, and neuroinflammation [59, 349,350]. It is assumed that these individual factors are responsible for the fact that the individual develops only one or a few specific diseases from a broad spectrum of possibilities resulting from the imbalance in physiology [63] (Fig. 1).

Aging significantly impacts chronobiology, including circadian rhythms. Older adults often experience changes in sleep architecture, such as reduced deep sleep and more fragmented sleep. The production and regulation of hormones like melatonin and cortisol, which are crucial for maintaining circadian rhythms, can diminish with age. This can lead to disrupted sleep-wake cycles and other physiological functions. Disruptions in circadian rhythms due to aging can contribute to various health issues, depression, CVDs, T2DM and neurodegenerative diseases such as Alzheimer's disease. Chronotherapy leverages these biological rhythms to optimize treatment efficacy and reduce side effects, offering a promising approach in several medical fields [351, 352].

Finally, lifestyle-related molecules and families of molecules such as oxysterols and AGEs, also play an important role in the development of age-related diseases [14-21].

The idea that the development of NCDs is based on a common pathogenetic pattern is supported by the fact that several NCDs often occur in one and the same person [multimorbidity] and that patients with one NCD are at risk of developing other NCDs [comorbidity] [353]. Numerous "unusual" combinations of comorbidities have been identified that can only be explained by a common underlying mechanism, such as OA and Parkinson's disease [354], glaucoma and Alzheimer's diseases [355], Alzheimer's disease and OA [356], glaucoma and COPD [357], COPD and cancer, CVDs and osteoporosis, Alzheimer's disease and psoriasis, COPD and osteoporosis, psoriasis and Alzheimer's disease, and psoriasis with CVDs, cancers, T2DM and Crohn’s disease [63], to name but a few.

### 6.1 Aging - a time of “harvest”?

Why do many chronic NCDs occur more frequently with increasing age? One of the main reasons is certainly that some diseases take longer to manifest themselves clinically. Chronic NCDs are generally characterized by long durations and slow progression. What we perceive as age-related diseases are therefore often the [late] manifestations of pathological processes whose development began much earlier in life as a result of an unhealthy lifestyle. As an example, fatty streaks, the first signs of incipient atherosclerosis, are already detectable in childhood and adolescence [358], long before clinical events occur. Hyperinsulinemia and IR, the prerequisites for T2DM, also develop long before the manifestation of diabetes. Osteoporosis develops gradually and can go unnoticed and without any recognizable symptoms in the early stages until it becomes noticeable in the form of bone fractures. It is also conceivable that the aging process contributes to the development of NCDs, for example by increasing susceptibility to diseases or pathologies [13].

### 6.2 Summary and perspectives

In summary, age-related diseases must be distinguished from the aging process itself, as all people age, but not all people develop age-related diseases. The development of age-related diseases is neither a universal phenomenon nor is it inevitable, as epidemiological data from HG societies show. Major differences between the exposome of the HG environment and that of the modern world plays a critical role. Lifestyle factors, in especially the WD and smoking, cause a dysregulation of the physiologic balance, which acts a “basic toolkit” for the development of many [most?] NCDs. It is believed that the interplay between this imbalance in physiology and "determinants" such as genetic susceptibility, biological variability, epigenetic changes and the microbiome is primarily responsible for which disease[s] [from a wide range of possibilities] develop[s] in an individual.

Ultimately, the "toolkit" theory represents an integration and extension of free radical and oxidative-inflammatory theories, as it assumes that OxS and inflammation are not the whole story, but that there is a broader disruption of physiological balance that includes not only the pattern described above, but also a number of other physiological factors, as outlined in a previous publication [63]. The proposed pathophysiological model offers new insights into the development of age-related NCDs and potential opportunities for prevention and intervention. An interesting approach would be to try to restore the physiological balance, e.g. by dietary measures in combination with [a bundle of] pharmacological interventions and lifestyle changes. Diets with a low glycemic load and a balanced ratio of omega-3 to omega-6 fatty acids, such as the Mediterranean diet and the Okinawa diet, as well as a Paleolithic diet should be considered.
